# Reference percentiles for FEV_1_ and BMI in European children and adults with cystic fibrosis

**DOI:** 10.1186/1750-1172-7-64

**Published:** 2012-09-07

**Authors:** Pierre-Yves Boëlle, Laura Viviani, Pierre-Francois Busson, Hanne V Olesen, Sophie Ravilly, Martin Stern, Baroukh M Assael, Celeste Barreto, Pavel Drevinek, Muriel Thomas, Uros Krivec, Meir Mei-Zahav, Jean-François Vibert, Annick Clement, Anil Mehta, Harriet Corvol

**Affiliations:** 1 AP-HP, Hôpital Trousseau - Pediatric Pulmonary Department, Hopital St Antoine – Public Health Department, Paris, France; 2 INSERM, U938, INSERM U707, Paris, France; 3 Université Pierre et Marie Curie – Paris6, Paris, France; 4 Dipartimento di Scienze Cliniche e di Comunità, Università degli Studi di Milano, Milan, Italy; 5 Cystic Fibrosis Centre Aarhus, Aarhus University Hospital, Aarhus N, Denmark; 6 Vaincre la Mucoviscidose, Paris, France; 7 Universitätsklinik für Kinder und Jugendmedizin, Tübingen, Germany; 8 Verona CF center, Verona, Italy; 9 Portuguese Registry for Cystic Fibrosis Collaborative Group, Lisbon, Portugal; 10 Department of Paediatrics, 2nd Faculty of Medicine, Charles University, Prague, Czech Republic; 11 Belgian Cystic Fibrosis Registry, Public health and Surveillance|, Scientific Institute of Public Health, Brussels, Belgium; 12 Unit for pulmonary diseases, University Children’s Hospital, University Medical Centre Ljubljana, Ljubljana, Slovenia; 13 Israeli National CF registry, Jerusalem, Israel; 14 Division of Medical Sciences, University of Dundee, Dundee, United Kingdom

**Keywords:** Cystic fibrosis, Forced expiratory volume in one second, Body mass index, Registry

## Abstract

**Background:**

The clinical course of Cystic Fibrosis (CF) is usually measured using the percent predicted FEV_1_ and BMI Z-score referenced against a healthy population, since achieving normality is the ultimate goal of CF care. Referencing against age and sex matched CF peers may provide valuable information for patients and for comparison between CF centers or populations. Here, we used a large database of European CF patients to compute CF specific reference equations for FEV_1_ and BMI, derived CF-specific percentile charts and compared these European data to their nearest international equivalents.

**Methods:**

34859 FEV_1_ and 40947 BMI observations were used to compute European CF specific percentiles. Quantile regression was applied to raw measurements as a function of sex, age and height. Results were compared with the North American equivalent for FEV_1_ and with the WHO 2007 normative values for BMI.

**Results:**

FEV_1_ and BMI percentiles illustrated the large variability between CF patients receiving the best current care. The European CF specific percentiles for FEV_1_ were significantly different from those in the USA from an earlier era, with higher lung function in Europe. The CF specific percentiles for BMI declined relative to the WHO standard in older children. Lung function and BMI were similar in the two largest contributing European Countries (France and Germany).

**Conclusion:**

The CF specific percentile approach applied to FEV_**1**_ and BMI allows referencing patients with respect to their peers. These data allow peer to peer and population comparisons in CF patients.

## Background

The outcome of cystic fibrosis (CF) patients has improved in recent decades, with mortality less than 5 percent during the first 10 years of life in patients treated with current multidisciplinary care [1]. However, disease severity remains variable among children, adolescents and adults [2,3]. In CF, severity is principally assessed by the decline in lung function because lung disease still remains the most common cause of death. Lung function in CF is almost universally calculated as percent predicted FEV_1_ referenced against a healthy population [4,5]. This particular choice reflects a widely held opinion that achieving normality remains the ultimate goal of CF care. Irrespective of whether or not this aspiration is achievable, a limitation of the current ‘reference against the normal range’ approach is that it does not provide a ranking of an individual patient’s status relative to age and sex-matched CF peers. Similar considerations apply to nutrition, which must be adequate to maintain lung function [6], or surrogate markers of disease severity calculated from semi-quantitative screening scales such as the Chrispin Norman Score that measures lung damage from chest radiography [7].

There have been attempts to overcome such limitations. Kulich and coworkers converted the absolute FEV_1_ into percentiles calculated from a registry of lung function values in North American CF patients [8]. In a related manner, McCormick and colleagues converted a chest x-ray severity score in childhood into population based percentiles [9]. Such self-referencing approaches, despite their calculation from cross sectional data, provide a reference base that allows the longitudinal tracking of CF disease outcome and informs on the relative position of a given patient against his or her peers.

As of now, the CF FEV_1_ specific percentiles have of necessity been obtained from US CF patients. It remains to be established whether these data are appropriate for European patients with CF. Indeed, there are many differences not only in how CF care is organized (for example, coverage of neonatal screening, timely referral to reference centers, variation in standards of care) but also in the environment between EU nations and across the Atlantic. Furthermore Kulich analyzed data from 15 years ago (1994 to 2001) that would not account for recent progress in CF care. To mitigate against such issues, we combined data from the European Cystic Fibrosis Society Patient Registry (ECFSPR) and data from the current French CF Modifier Gene Study, to obtain CF reference percentile equations for lung function and body mass index that would apply to current European CF patients and would additionally provide the ranking of an individual patient’s status relative to age and sex-matched CF peers. As a second objective, we examined whether FEV_1_ and BMI in CF patients differed between European countries and across two continents, Europe and North America (USA).

## Methods

### Patients

We used the multinational ECFSPR as primary source of data and additional observations from the French CF Modifier Gene Study (MUCONAT). The former was set up to “measure, survey and compare CF aspects and treatments” in European countries [3,10]. Fourteen countries contributed data to the present study (Austria, Belgium, Bulgaria, Czech Republic, Denmark, France, Germany, Greece, Israel, Italy, the Netherlands, Portugal, Slovenia, Sweden). The coverage of the study relative to the overall CF population in a given country was estimated using genetic prevalence estimates reported by Farrell et al. [11], by Efrati et al. [12] for Israel, and by the French CF national registry for France. Patients’ consent was obtained from every participating country and all the registry protocols were compliant with the relevant national data protection laws. ECFSPR data at a patient level were collected on a anonymous basis.

The MUCONAT project collects data on prevalent and incident CF cases. It was approved by the French ethical committee (CPP n°2004/15) and the information collection was approved by the CNIL (n°04.404). As for the ECFSPR, the MUCONAT data were collected on a strictly anonymous basis. The project is based on the participation of 38 out of the 49 French CF centers. Prospective data collection started in 2007 for all prevalent and incident cases.

The following information was extracted from both databases: FEV_1_ measurements (in L), country of residence, *CFTR* genotype, sex, height, weight and age, BMI. The ECFSPR covered the period 2004 to 2007, with one observation per year and per patient. Data for years 2008 to 2010 were obtained for French patients from the MUCONAT database, so that there was no overlap with the ECFSPR source. The same patient may have contributed data over several years; however the current ECFSPR did not allow reliable data linkage throughout the years for all the participating countries, so longitudinal aspects were not taken into account. Measurements in patients after lung transplantation were removed for all the analyses.

### Statistical analysis

The FEV_1_ percent predicted (FEV_1_ pp) were estimated according to the Knudson equations [5], and the BMI z-scores were computed using the WHO 2007 standards [13].

In data contributed to the ECFSPR, some countries reported the “best” annual FEV_1_ measurement, while others, including the 2 largest contributing countries (France and Germany), reported an unselected measurement. Using the best measurement for computation leads to overestimation in the CF specific percentile curves and limit its use to assess patients from unselected measurement. To limit such bias, we transformed “best” FEV_1_ values before calculation (*see* Additional file 1). In short, the correction was computed as follows: using the French data, where a systematic longitudinal collection of all FEV_1_ values is carried out, we determined, by sex and age, the average difference between the “best” annual FEV_1_ and an unselected value of the same year. A corrected FEV_1_ measurement was then obtained by subtracting this value from the reported FEV_1_ value in countries reporting best values. As a sensitivity analysis, we also analyzed the data without correction. This correction was not required for BMI, as the reported data was not selected.

Quantile regression was used to estimate CF-specific reference equations for FEV_1_ and BMI. The *q*-quantile (or *q*-percentile) in a given distribution is the value below which the smallest q percent of the population is found: for example, the median is the 50^th^ quantile. Quantile regression allows modeling of quantile values as a function of covariates [14], whereas standard regression only models the mean value. Here, we modeled quantiles of the FEV_1_ and BMI distribution according to age and height in European CF patients, separately in men and women. We used cubic B-splines to capture the non-linear dependence of FEV_1_ on age and height, using 6 nodes to avoid overfit [8]. FEV_1_ percentiles from 1 to 99% were fitted as a function of age alone, height alone and of age and height together. BMI quantiles were estimated as a function of age. Confidence intervals for quantiles were obtained using the bootstrap.

The difference between the European and the USA FEV_1_ CF specific percentiles and between the CF-specific BMI percentiles and the WHO normative values was assessed by the difference in area under the curve with a bootstrap test. Inter country variation was assessed between the 2 countries contributing the most patients (France and Germany), one group consisting of smaller countries with large coverage (> 75%) of CF patients (Israel, Denmark, Belgium, the Netherlands, Slovenia and Czech Republic) and a 4^th^ group with other countries (Bulgaria, Sweden, Portugal, Greece, Austria, Italy). Confidence intervals for the median percentile in each country was obtained using bootstrap and corrected for multiple comparisons by the Bonferroni rule (4 groups times 3 age classes). All analyses were done using the R software v2.14 (quantreg version 4.62).

## Results

### Demographic description

A summary of lung function (FEV_1_) and nutritional (BMI) parameters is presented in Table 1 for all CF patients. The median national coverage was 74%, with a large range between countries (from 15% to >99%). Overall, we used 34859 measurements of FEV_1_ in the age range 6 to 40 years, and 40947 BMI measures in the age range 0 to 40 years, corresponding to 16781 patients. The median female to male ratio was 0.92 (range in the countries: 0.7 to 1.3) and decreased with age, from 0.93 in the <10 years old to 0.82 in the >35 year olds (Chi-squared test for trend, P < 0.0001). The number of measurements in adults (> 20 years old) represented 40% of all reports, with little variation among countries, except for Bulgaria and Slovenia where only pediatric cases were available. Median percentage of p.Phe508del homozygosity was 47.8%, although with a wide range from 14% in Israel to 83% in Hungary [15].

**Table 1:** Demographic description

**Country**	**CF patients**	Estimated Coverage^*^	**Number of measurements**	**Sex-ratio (F)**	**p.Phe508del homozygosity**	**Age (yrs)**	Adults**	**FEV**_**1**_percent predicted (%)***	**BMI z-score**
	**(n)**	**(%)**	**(n)**	**(%)**	**(%)**	**Mean [range]**	**(%)**	**Mean ± SD**	**Mean ± SD**
ECFS Patient Registry (2004-2007)									
France	5147	89%	14674	47.9	48%	16.7 [0 - 78.4]	34%	74.4 ± 31.8	-0.63 ± 1.07
Germany	5039	74%	19272	48.1	64%	18.1 [0 - 68.2]	39%	75.0 ± 31.6	-0.54 ± 1.09
High coverage countries	3671	93%	9213	47.4	53%	18.6 [0 - 77.4]	41%	80.1 ± 28.4	-0.35 ± 1.08
Low coverage countries	1347	18%	3774	48.3	44%	18.3 [0 - 69.0]	40%	81.32 ± 28.0	-0.21 ± 1.08
MUCONAT (2008-2010)									
France	1577	39%	3549	47.9	51.8%	20.3 [6.0 - 40.0]	47%	72.9 ± 31.2	-0.53 ± 1.00
Overall	16781	-	50482	47.9	47.8	18.0 [0 - 78.4]	39%	75.7 ± 31.1	-0.52 ± 1.07

* Estimated Coverage derived from prevalence estimates reported by Farell et al., Efrati et al. and the French CF national registry.

** Age > 20 years.

*** FEV_1_ percent predicted calculated with Knudson equation.

### Lung function

**Figure 1:**
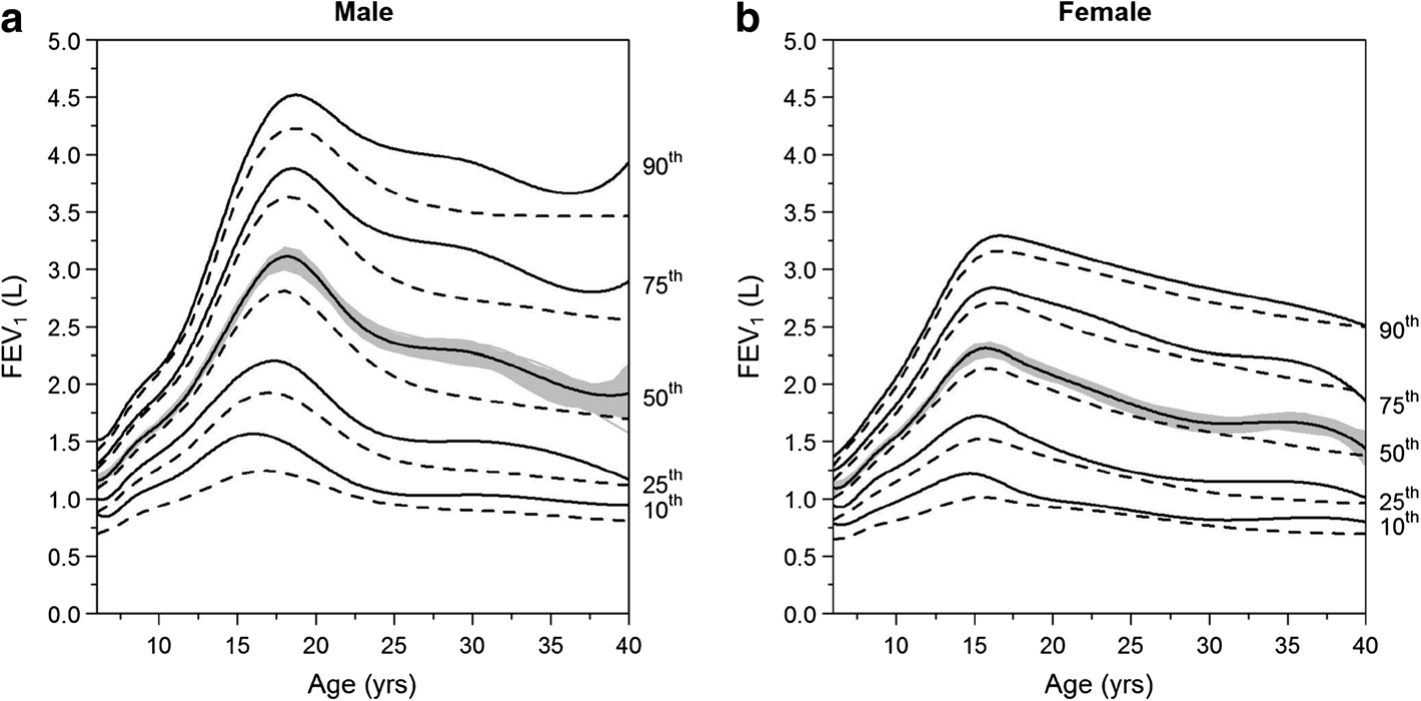
**Cystic fibrosis specific FEV**_**1**_**percentiles according to age and sex, in males (a) and females (b).** The grey zone shows the 95% confidence interval for the median percentile; and the dashed lines correspond to reference values obtained in US patients.

The mean FEV_1_ pp showed a decline across age groups, from 95% for ages 6-13 years and 76% for ages 13-20 years to 61% for ages 20-40 years. The CF specific FEV_1_ percentiles according to age, sex and height are shown in Figure 1. As expected, the median FEV_1_ increased throughout childhood (almost linearly) and decreased thereafter. Starting from approximately 1 L at age 6, irrespective of sex, the median FEV_1_ increased up to 3 L in boys at age 18 and to 2.3 L in girls at age 16. For both sexes, the interquartile range was the largest at the peak, spanning from 2.2 to 3.7 in boys at age 18, and 1.7 to 2.7 in girls at age 16. The median FEV_1_ increased monotonously with height, by approximately 0.25 L with each additional 10 cm (data not shown).

As shown in Figure 1, we found differences between the European and the USA percentiles. For example, a 20 year old European male patient 170 cm of height, with an FEV_1_ of 3 liters ranked at the 65^th^ percentile among US CF patients, but only at the 61^st^ percentile among European CF patients. The CF percentiles values according to age or height were higher than those obtained in the US at the first, second and third quartiles (P < 0.001 for comparison at each quartile). However, the overall topography of the quantiles paralleled that of the US with an upwards offset. The correlation was excellent between the US- and the European-calculated age and height adjusted CF specific percentiles (r = 0.99, P < 0.001).

Differences between US and European percentiles were more pronounced in males (the median increased by 0.2 L on average) than in females (increased by 0.1 L). The difference in median FEV_1_ between Europe and the US was also larger in older patients: 0.1 L difference in the < 15 years old but 0.3 L in males > 15 years and 0.15 L in females. Finally, among the young patients (<15 years old), the difference was greater in the 10^th^ percentile than in the 90^th^ percentile: in the latter, the curves were almost the same between the EU and the US.

### Nutritional outcome (BMI)

**Figure 2:**
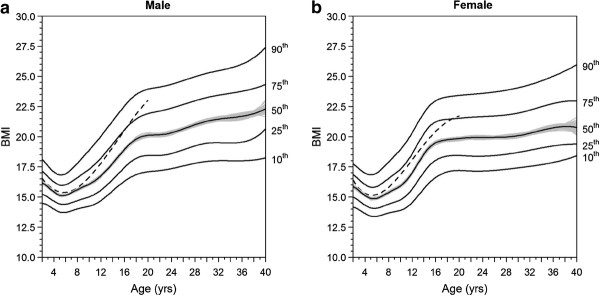
**Cystic fibrosis specific BMI percentiles according to age, in male (a) and female (b) patients.** The grey zone shows the 95% confidence interval for the median percentile. The dashed line shows the normative median from the WHO 2007 definition.

Quantiles of BMI according to age are shown in Figure 2 for males and females. The CF specific BMI profiles with age were typical of BMI growth curves, but were lower than the WHO 2007 [16] normative values at all ages, with important sex differences. For CF boys, the median BMI remained close to the WHO reference up to age 10 (i.e. less than 2.5 % difference for the median). In CF girls, the nutritional status was already impaired by age 8. The difference to the WHO normative values was more pronounced in boys than in girls in adolescents and young adults. Overall, at age 20, only one quarter of young adults with CF were above the median normative value.

### Inter-country comparisons

**Figure 3:**
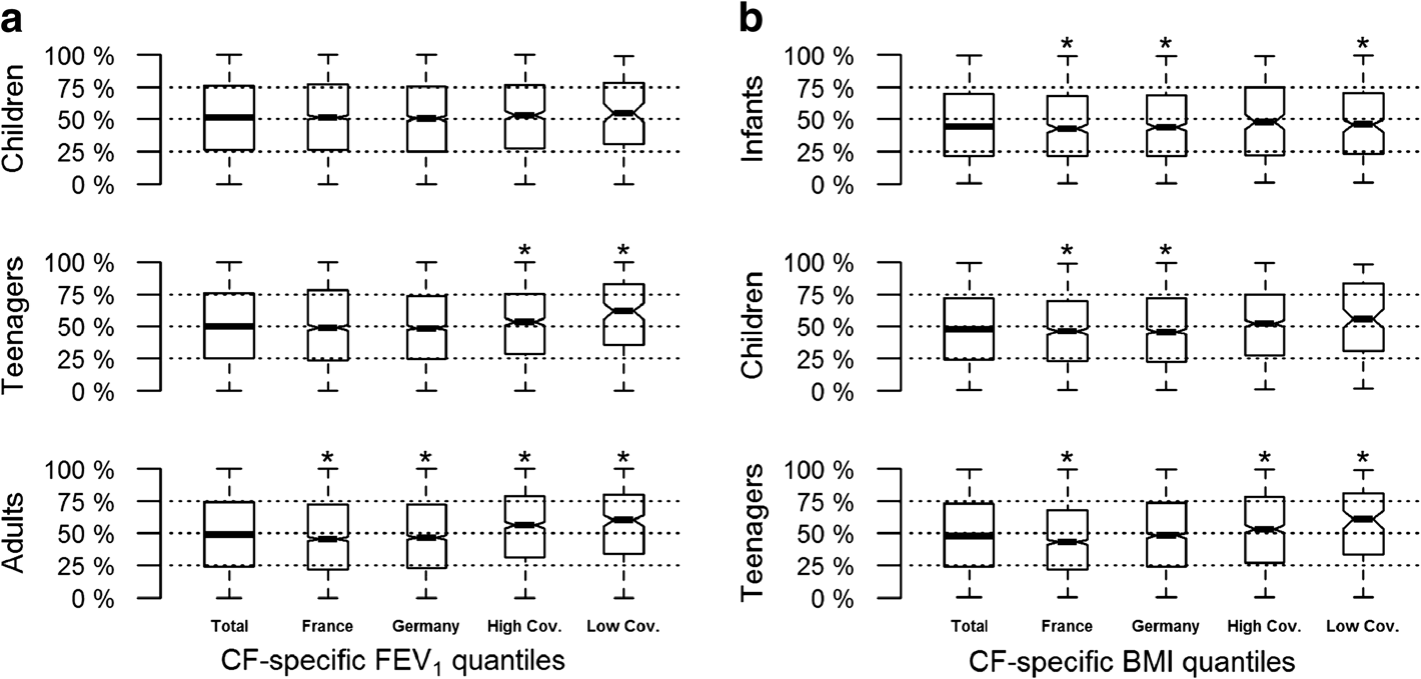
**Age and country cystic fibrosis specific FEV**_**1**_**(a) and BMI (b) percentile distributions in European cystic fibrosis patients.** Boxes extend from the first to the last quartile,with the median as a thick line, and whiskers over the whole range. Notches correspond with the 95% Bonferroni adjusted (12 comparisons) confidence intervals for the country median, a star indicating that the expected median percentile (50%) is not within the confidence interval. Definitions*:* Infants: 2 to 6 years old; Children: 6 to 13 years old; Teenagers: 13 to 20 years old; Adults: over 20 years old.

Figure 3 presents both the age and height adjusted FEV_1_ and age adjusted BMI percentiles by country and age group. Importantly, there was no major variation in the FEV_1_ CF percentile distributions according to participating countries (Mann–Whitney-Wilcoxon test for France and Germany, P = 0.5). For example, the median FEV_1_ age and height adjusted CF percentile among all patients was 49% in France and Germany and 52% in other countries, all very close to the expected 50%. In France and Germany, the country-level median percentile was close to 50% in all age classes although somewhat below in adults (respectively 45% and 47% for France and Germany). In other countries, the observed medians in the 3 age classes were always slightly larger than 50%. Moreover, the observed median percentile in each country was not correlated with either sample size (P = 0.4), coverage (P = 0.4) or percentage of p.Phe508del *CFTR* homozygosity (P = 0.4). Similar results were found for p.Phe508del homozygous patients (data not shown). The situation was similar for BMI with no discernible pattern across countries.

## Discussion

Achieving normality in lung function or nutritional status is the ultimate goal of CF care, even if the possibility of achieving this ideal remains a moot point given the multisystem nature of CF and the complexities of the variable genotype-phenotype relationship [17]. Using a healthy population as a reference (as is universal with FEV_1_ pp) determines how far CF patients lie from normality. We propose here that additional information may be gleaned by referencing CF patients against age and sex matched CF peers. A large database of European cystic fibrosis patients was used to compute these reference percentiles and the software code is available from the authors on request.

As of today, such CF-specific reference- ranges have mainly been used for research purposes, for example to provide a quantitative phenotype for comparing patients [18]. However, they could also be useful in a given clinic to either directly compare the performance between CF centers or to help homogenize patients for inclusion in clinical trials. This disease-referencing approach could also be useful to physicians and patients to help visualize the status of a single patient relative to his or her peers. For example, a longitudinal plot could highlight unusual worsening of clinical conditions within European CF framework and help the physician on the need for more aggressive therapies.

One may worry that referencing CF patients relative to their peers could lessen physicians’ efforts to improve CF patients’ health status, if they were satisfied with their patients’ progress referenced against their peers. In other words, care must be taken in interpretation such that the CF specific percentiles should not be interpreted as “normative” values, but, as discussed above, as a reference providing additional information. Reassuringly, there was no indication that the availability of such equations from US patients since 2005 negatively impacted CF care as also evidenced by the approach of McCormick and colleagues who calculated similar centiles for chest X-ray scores. Furthermore, our local patient representatives, as required by our consents and ethical practices on the use of such data, when shown the approach, spontaneously remarked that they would like to know how they were performing relative to others in Europe. Future studies will have to determine the impact of providing both types of reference information, for example in the case of specific CFTR variants such as G551D-CFTR which now has a new therapy [19].

Of necessity, it will be imperative to regularly update the CF specific reference equations to reflect CF care improvement. For example, it was reassuring that the European CF-specific FEV_1_ percentiles were greater than those computed a few years ago from US CF patients [8]. This difference was more pronounced in the lower percentiles at all ages. It is unlikely that this difference results of differential mortality, as mortality curves are very similar for CF patients in large European countries and the US [20,21]. Better organization of CF care over time may have led to a larger number of less severe patients included in registries, therefore leading to improved overall performance. However, it remains equally likely that changes in the efficacy of CF care over time is an explanation for the observed differences given that almost one decade passed between data collection in the US and the European studies. Significant improvement in the survival and clinical status of CF patients has been achieved during this time, by earlier CF diagnosis, better nutritional support and mucus drainage, and better diagnosis and treatment of CF-related complications [22]. An argument in favor of the increased care efficacy is that the improvement was more pronounced in the patients with the poorest lung function. In addition, the median CF specific FEV_1_ percentile calculated in today’s North-American patients using the Kulich equations tends to be above the expected 50^th^ percentile [18].

Using the ECFSPR data permitted analysis of a large number of measurements obtained from several European countries. Participation to the ECFSPR is on a voluntary basis, and the coverage (i.e. the proportion of CF living population actually included in the registry) ranges from 15% to >99%, with 9 countries having coverage greater than 50%. Little selection bias is expected in countries with large coverage, while it may be significant in countries where participation is limited to some voluntary CF centers. In the latter case, the extent to which the reported patients’ characteristics are biased relative to the whole country CF population is unpredictable. However, in our analysis, the estimates were not substantially affected when we excluded countries with small coverage.

We computed the CF specific equations so that unselected data could be referenced against these curves. The FEV_1_ values reported from countries where only the best measurement was provided were therefore corrected before analysis. Otherwise, it could have been the case that the reference curves overestimate the true status of the CF patients, as a consequence of analyzing mostly best measurements. However, additional analysis of the raw data, without corrections, yielded identical results, showing that the impact was overall small (see supplemental material). Thus the common assumption that selective reporting of best lung function can confound data interpretation is not supported by our findings.

One other result of this study is that the European CF BMI percentiles were in good agreement with the normative WHO 2007 curves up to age 7 but lower thereafter [16]. Despite recommendations to achieve greater fat and calorie intake, CF children and teenagers typically consume similar nutritional amounts as their healthy peers [23,24]. A positive association has been observed between a better nutritional status and a higher pulmonary function, with an inverse relation to morbidity and mortality [6,25]. The BMI of CF females remained closer to the WHO reference charts, while it has been reported that they experience steeper trajectories of health decline [26,27].

In the inter-country comparison, we conjoined countries with a small number of patients and large coverage, and all countries with low coverage. This was done to limit the effect of chance variation that could arise from small populations. The outcomes in FEV_1_ were somewhat larger in countries with small coverage. As it is not possible to rule out selection bias such as a survivor effect coupled to under diagnosis in these countries, this result should not be taken as evidence of better outcomes. The BMI inter-country comparison was less affected by coverage.

Although the FEV_1_ CF specific percentiles provide a useful approach for comparing CF patients to their peers, they do not correct for attrition due to mortality [18]. For example, the population median CF percentile is 50% at all ages, but it is obvious that ranking 50^th^ among 8 years old CF patients is different from ranking 50^th^ at age 40. The use of “survival adjusted” CF specific percentiles has been proposed to measure severity as a quantitative trait irrespective of age [18]. More data will be required to fully adapt this method to the European situation which should become easier as neonatal screening takes hold across Europe thus significantly reducing ascertainment bias. In the meantime, the excellent correlation of the percentiles found from the European and the US analyses will in future allow cross comparison with US derived “common” phenotype in international studies involving North-America and Europe. This was indeed a critical limiting factor in earlier analyses [28,29].

## Conclusions

To conclude, although achieving normality is the ultimate goal of CF care, separately referencing against age and sex matched CF peers provides additional information to compare CF populations and better illustrates the range of variability between patients. These new reference equations also provide tools for computing quantitative traits for use in genome wide analyses [30]. Ours is only a first step towards the possibility to a fair comparison of European CF patients and health system performance. With the future availability of large phenotypic databases, it might be possible to apply our approach in other rare diseases, an emerging priority across the globe [31].

## Competing interests

The authors declare that have no competing interests.

## Authors’ contributions

PYB and HC designed the study and wrote the manuscript. PYB and PFB analysed the data. AM, LV and HO have been involved in the conception and design of the study, in the acquisition and interpretation of the data and in critically revisiting the manuscript. SR, MS, BA, CB, PD, MT, UK, MMZ, JFV and AC have been involved in the acquisition of the data and in revisiting the manuscript. All authors read and approved the final manuscript.

## Supplementary Material

Additional file 1 Table S1.Mean difference between *Best* FEV_1_ measurement of the year and *Unselected* FEV_1_ measurement, according to sex and age. Click here for file
