# Supplementary material for: Reference percentiles for FEV1 and BMI in European children and adults with cystic fibrosis
Source: Orphanet J Rare Dis. 2012 Sep 7;7:64. doi: 10.1186/1750-1172-7-64 (PMC3520808; doi:10.1186/1750-1172-7-64)

# Additional File

# Reference percentiles for FEV_1_ and BMI in European children and adults with Cystic Fibrosis

Pierre-Yves Boëlle^1,2^, Laura Viviani^3^, Pierre-François Busson^1^, Hanne V Olesen^4^, Sophie Ravilly^5^, Martin Stern^6^, Baroukh M. Assael^7^, Celeste Barreto^8^, Pavel Drevinek^9^, Muriel Thomas^10^, Uros Krivec^11^, Meir Mei-Zahav^12^, Jean-François Vibert^1,2^, Annick Clement^2,13^, Anil Mehta^13^, Harriet Corvol^2,13^ on behalf of the French CF Modifier Gene Study Investigators and the European CF Registry Working Group

**Institutions:**

1. AP-HP, Hôpital St Antoine, Biostatistics Department ; Inserm UMR-S707, Paris, France

2. Université Pierre et Marie Curie – Paris 6, Paris, France

3. Dipartimento di Scienze Cliniche e di Comunità, Università degli Studi di Milano, Milano, Italy

4. Cystic Fibrosis Centre Aarhus, Aarhus University Hospital, Aarhus N, Denmark

5. Vaincre la Mucoviscidose, Paris, France

6. Universitätsklinik für Kinder und Jugendmedizin, Tübingen, Germany

7. Verona CF center, Verona, Italy

8. Portuguese Registry for Cystic Fibrosis Collaborative Group, Lisbon, Portugal

9. Department of Paediatrics, 2nd Faculty of Medicine, Charles University, Prague, Czech Republic

10. Belgian Cystic Fibrosis Registry, Public health and Surveillance, Scientific Institute of Public Health, Brussels, Belgium

11. Unit for pulmonary diseases, University Children’s Hospital, University Medical Centre Ljubljana, Ljubljana, Slovenia

12. Israeli national CF registry, Israel

13. Division of Medical Sciences, University of Dundee, Dundee, United Kingdom

6. AP-HP, Hôpital Trousseau, Pediatric Pulmonary Department; Inserm U938, Paris, France

# Supplementary Material and Methods

**Statistical analysis**

**Correcting bias introduced by FEV_1_ measurement selection**

Some countries participating to the ECFSPR recorded an unselected FEV_1_ measurement every year (e.g the last one, or the one closest to the patient's birthday, etc.), whereas other countries reported the best FEV_1_ measurement of the year. This is likely to bias upwards the overall performance of patients. In order to correct for this bias, the difference between the best measurement and an unselected measurement was evaluated using data from the French patient cohort, for which a systematic reporting of FEV_1_ measurements is under way.

As expected, best FEV_1_ measurements were slightly larger than unselected measurements: the mean difference was 0.14 (SD 0.25) in male patients and 0.12 (SD 0.2) in female patients (p<0.001). The difference was also linked to age (p<0.001 for male and female), as shown in **figure S1**.

The expected difference between an unselected and the best measurement was computed as a function of age. The data from countries reporting the “best” measurement was then corrected by subtracting the mean difference from the reported FEV_1_ according to age and sex (**table S1**), thus improving the inter-country comparability.

The correction had however little impact on the computed quantiles (**figure S2**): the median difference between the quantiles computed using non-corrected values and the quantiles computed using corrected values was 0.01 (Q1-Q3 [0 - 0.01]) for both sexes.

**Table S1:** Mean difference between *Best* FEV_1_ measurement of the year and *Unselected* FEV_1_ measurement, according to sex and age.

| **Female** | |  | **Male** | |
| --- | --- | --- | --- | --- |
| Age | Mean difference |  | Age | Mean difference |
| 6 | 0,0097 |  | 6 | 0,0130 |
| 7 | 0,0481 |  | 7 | 0,0519 |
| 8 | 0,0591 |  | 8 | 0,0614 |
| 9 | 0,0645 |  | 9 | 0,0714 |
| 10 | 0,0679 |  | 10 | 0,0785 |
| 11 | 0,0782 |  | 11 | 0,0988 |
| 12 | 0,0748 |  | 12 | 0,1011 |
| 13 | 0,0967 |  | 13 | 0,1056 |
| 14 | 0,1041 |  | 14 | 0,0971 |
| 15 | 0,1849 |  | 15 | 0,1149 |
| 16 | 0,1670 |  | 16 | 0,1352 |
| 17 | 0,1655 |  | 17 | 0,1933 |
| 18 | 0,1841 |  | 18 | 0,2044 |
| 19 | 0,1763 |  | 19 | 0,2305 |
| 20 | 0,2057 |  | 20 | 0,2241 |
| 21 | 0,1607 |  | 21 | 0,2286 |
| 22 | 0,1791 |  | 22 | 0,2321 |
| 23 | 0,1483 |  | 23 | 0,2282 |
| 24 | 0,1820 |  | 24 | 0,1960 |
| 25 | 0,1247 |  | 25 | 0,1774 |
| 26 | 0,1453 |  | 26 | 0,1735 |
| 27 | 0,1878 |  | 27 | 0,1655 |
| 28 | 0,1376 |  | 28 | 0,1876 |
| 29 | 0,1273 |  | 29 | 0,1867 |
| 30 | 0,1658 |  | 30 | 0,1466 |
| 31 | 0,1352 |  | 31 | 0,1431 |
| 32 | 0,1310 |  | 32 | 0,1598 |
| 33 | 0,1387 |  | 33 | 0,1572 |
| 34 | 0,1142 |  | 34 | 0,1718 |
| 35 | 0,1358 |  | 35 | 0,1427 |
| 36 | 0,1289 |  | 36 | 0,1143 |
| 37 | 0,1225 |  | 37 | 0,1206 |
| 38 | 0,0967 |  | 38 | 0,1240 |
| 39 | 0,1292 |  | 39 | 0,1192 |
| 40 | 0,0930 |  | 40 | 0,1774 |

**Figure S1**: Difference between *Best* FEV_1_ measurement of the year and *Unselected* FEV_1_ measurement, according to sex and age.


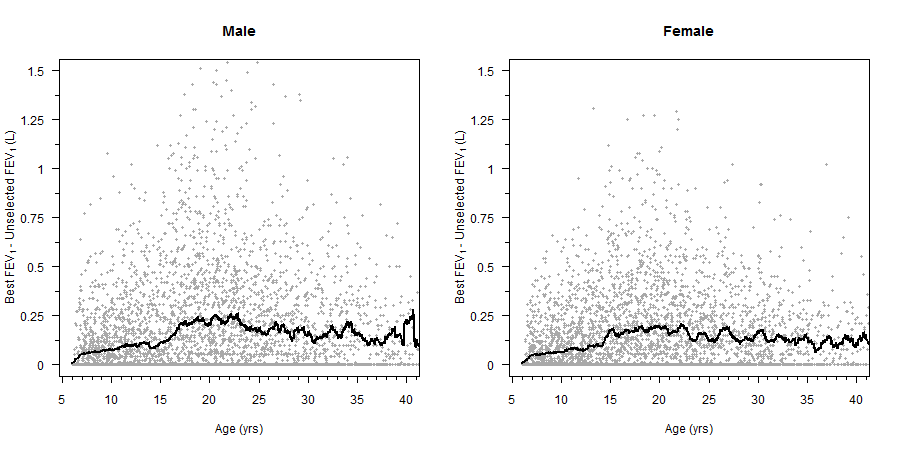


**Figure S2**: Cystic fibrosis specific FEV_1_ percentiles according to age, in male (left) and female (right) patients, before (solid line) and after (dashed line) correction of the difference between *Best* and *Unselected* FEV_1_ measurement.


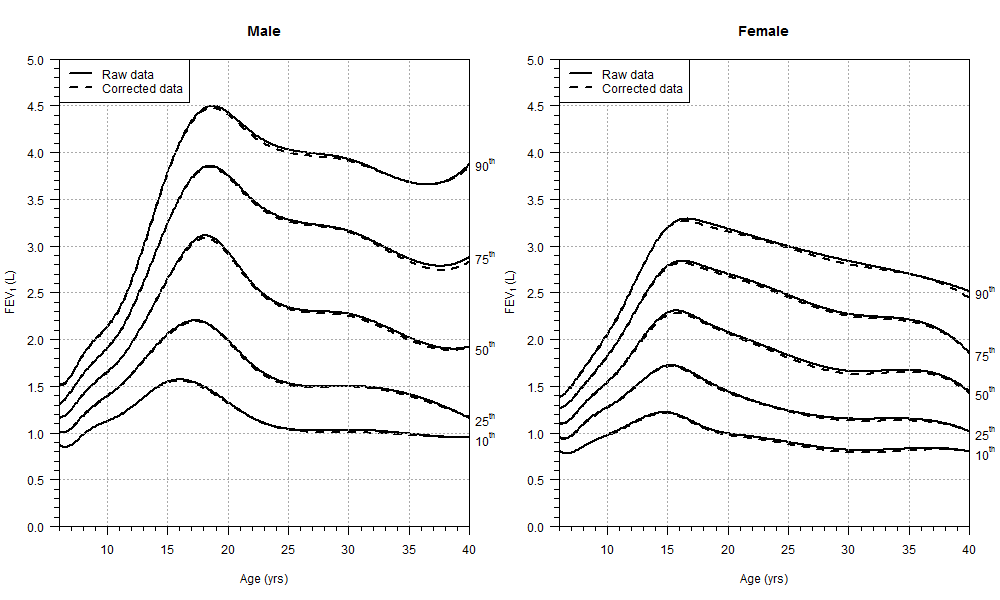

Supplement: Additional file 1 — Table S1.Mean difference between Best FEV1 measurement of the year and Unselected FEV1 measurement, according to sex and age. [file 1750-1172-7-64-S1.doc]
